# VTag: a semi-supervised pipeline for tracking pig activity with a single top-view camera

**DOI:** 10.1093/jas/skac147

**Published:** 2022-04-29

**Authors:** Chun-Peng J Chen, Gota Morota, Kiho Lee, Zhiwu Zhang, Hao Cheng

**Affiliations:** Department of Animal Science, University of California, Davis, CA 95616, USA; Department of Animal and Poultry Sciences, Virginia Polytechnic Institute and State University, Blacksburg, VA 24061, USA; Center for Advanced Innovation in Agriculture, Virginia Polytechnic Institute and State University, Blacksburg, VA 24061, USA; Division of Animal Sciences, University of Missouri, Columbia, MO 65211, USA; Department of Crop and Soil Sciences, Washington State University, Pullman, WA 99164, USA; Department of Animal Science, University of California, Davis, CA 95616, USA

**Keywords:** computer vision, pig activity, object tracking, RGB camera

## Abstract

Precision livestock farming has become an important research focus with the rising demand of meat production in the swine industry. Currently, the farming practice is widely conducted by the technology of computer vision (CV), which automates monitoring pig activity solely based on video recordings. Automation is fulfilled by deriving imagery features that can guide CV systems to recognize animals’ body contours, positions, and behavioral categories. Nevertheless, the performance of the CV systems is sensitive to the quality of imagery features. When the CV system is deployed in a variable environment, its performance may decrease as the features are not generalized enough under different illumination conditions. Moreover, most CV systems are established by supervised learning, in which intensive effort in labeling ground truths for the training process is required. Hence, a semi-supervised pipeline, VTag, is developed in this study. The pipeline focuses on long-term tracking of pig activity without requesting any pre-labeled video but a few human supervisions to build a CV system. The pipeline can be rapidly deployed as only one top-view RGB camera is needed for the tracking task. Additionally, the pipeline was released as a software tool with a friendly graphical interface available to general users. Among the presented datasets, the average tracking error was 17.99 cm. Besides, with the prediction results, the pig moving distance per unit time can be estimated for activity studies. Finally, as the motion is monitored, a heat map showing spatial hot spots visited by the pigs can be useful guidance for farming management. The presented pipeline saves massive laborious work in preparing training dataset. The rapid deployment of the tracking system paves the way for pig behavior monitoring.

## Teaser

The presented pipeline, VTag, saves massive laborious work in preparing labeled training datasets and setting up environment for pig tracking tasks. VTag can be deployed rapidly and paves the way for pig behavior monitoring.

## Introduction

Precision livestock farming, which collects detailed measurements of animals through sensors or cameras, has become an important research focus with the rising demand of animal production (Morota et al., 2018; Clark et al., 2020). Monitoring animal activity can facilitate the management of animal production, and it is conventionally conducted by frequently visiting the farms or manually reviewing recorded videos (Larsen et al., 2019). However, these approaches can be subjective and laborious. Alternatively, the technology of computer vision (CV), which is inspired by human vision that can intuitively focus on objects of interest and exclude noisy signals, can automate monitoring animal activity solely based on information obtained from video recordings. Automation is fulfilled by deriving imagery features from a series of computational tasks, such as video segmentation and edge detection, guiding the CV models to recognize animals’ body contours, positions, and behavioral categories.

With the CV technology, many studies have shown promising results in practicing precision livestock farming. For example, it used to be costly to manage cattle in large-scale pasture lands. Coupling with unmanned aerial vehicles, CV is possible to automate cattle counting in real-time with labor costs substantially reduced (Xu et al., 2020). In smaller-scale indoor farms, CV systems were also used to detect body cleanness (Li et al., 2019a), entirety (Fang et al., 2020), structure (Liu et al., 2020), and behaviors (Okura et al., 2019; Fuentes et al., 2020; Ren et al., 2021) for animal production. This technology is particularly beneficial to the swine industry, as pigs are usually group-housed in indoors settings. By deploying one top-view RGB colors camera, producers can track pig activity by capturing their positions and identities in a high-throughput manner (Yang et al., 2018; Li et al., 2019b; Zhang et al., 2019; Huang et al., 2020). To assess complicated traits that are labor intensive to be measured, the deployment of multiple cameras or RGB-D depth sensing cameras can provide extra dimensions of information to enhance the CV system. Many successful applications have also demonstrated automation in assessing body weight (Yu et al., 2021), feeding behaviors (Leonard et al., 2019), and more precise measurement of real-time pig positions (Tu et al., 2020) in recent years.

Nevertheless, challenges still exist in the current CV systems and make CV difficult to be widely implemented in most farming environments. First, the performance of CV systems is sensitive to the imagery features, which are derived based on the observed imagery patterns under certain environmental conditions (e.g., lighting). When the CV system is deployed in a new environment, its performance may decrease as the features are not generalized enough to be associated with pig morphological patterns under different illumination conditions (Chen et al., 2021). Second, most CV systems are established by supervised learning, in which intensive effort in labeling ground truths for the training process is required. The insufficient size or quality of training datasets may be a harmful factor for the model robustness. Lastly, many successful CV systems are built by deep learning models, in which tens of millions of unknown parameters are estimated (He et al., 2015; Simonyan and Zisserman, 2015; Bochkovskiy et al., 2020). Such nature makes CV systems challenging to implement, limiting a wider deployment of high-throughput monitoring in animal industry.

Due to the difficulties described in the current CV approaches, tracking pig activity is a challenging task without considerable labor efforts. The objective of this paper is to develop a semi-supervised pipeline, Virtual Tag (VTag), to automate long-term tracking of group-housed pigs. In this pipeline, successful tracking algorithms (hereafter trackers) are implemented. They include Sparse Optical Flow proposed by Lucas and Kanade (LK) (Lucas and Kanade, 1981), multiple instance learning (MIL) (Babenko et al., 2009), and channel and spatial reliability (CSRT) (Lukežič et al., 2018) that learn representatives from the object of interest and to find the similar image region in the next input video frame. These algorithms are lightweight and require no specific computing resources such as graphics processing units (GPU). The implemented tracker substantially reduce efforts in labeling pig positions for every single frame. To start tracking, users can either assign initial positions, or VTag can predict the positions based on their motion, which is anticipated to be effective features under different monitoring environments. We validated VTag by four three-hundred-frame videos collected from our farming trials, and the benchmark test is performed to compare the performance and detected frames per second (FPS) of the implemented trackers and other state-of-the-art models, such as YOLOv5 (Jocher et al., 2022) and Mask R-CNN (He et al., 2017). In addition, VTag is released as a friendly software tool in both a graphical user interface (GUI) and a Python library, allowing users to freely utilize the labeled data for their following research. Therefore, neither hard-coded features selected by human experts nor large training datasets labeled from a massive manual work are required in our pipeline. The complete algorithm and source code are available at https://github.com/vt-ads/vtag.

## Materials and Methods

### Data acquisition

All animal experiments were approved and carried out in accordance with the Virginia Tech Institutional Animal Care and Use Committee (IACUC) under protocol #19-182. The demonstrated video recordings were obtained from (Yu et al., 2021), which reported the image-based live body weight prediction of non-restrained grower pigs. The pigs entered the trial at 5 wk post-weaning. The imaging system was built with a laptop-controlled camera (Intel RealSense D425) that captured RGB and depth videos with resolution of 848 × 480 pixels. The camera was installed at a height of 2.25 m perpendicularly to the floor in each 5 × 7 ft pen, where pigs can freely move and walk during the entire recording. In each day, each monitored pen was recorded in a three-hundred-frame video at a rate of 6 frames per second. Raw videos were saved in Robot Operating System bag video format, and the decoder Intel RealSense Viewer was applied to obtain sequential image files as the input data. In this study, only RGB-converted grayscale images were used, and depth and color information were excluded from the pipeline. Each video clip had 300 timeframes. There were four video clips being evaluated for the performance of the presented pipeline: 3 clips contain 1, 2, and 3 pigs (denoted as 1-pig, 2-pig, 3-pig datasets in the following paragraphs), respectively. The last clip also contains 2 pigs, but more motion was observed (denoted as 2-pig (high motion)).

### Implemented trackers

In VTag, 3 trackers, LK, CSRT, and MIL, are implemented by Python OpenCV (Bradski, 2000). To simplify the pipeline, users only need to provide 2 parameters to the trackers. First, positions coded in (*x*, *y*) coordinates are required to be set to start the tracking task. Each pig needs to be assigned to at least one starting position. The position can be provided automatically by VTag, which uses detected motion to propose starting positions for users. The detailed process is described in the following section. The second parameter is the size of the bounding box that covers the tracking area. The bounding box is centered on the starting position assigned from the first parameter, and it is desired to cover diverse textures (e.g., contour edges) for the best tracking performance.

### Motion detection

**Figure 1.**
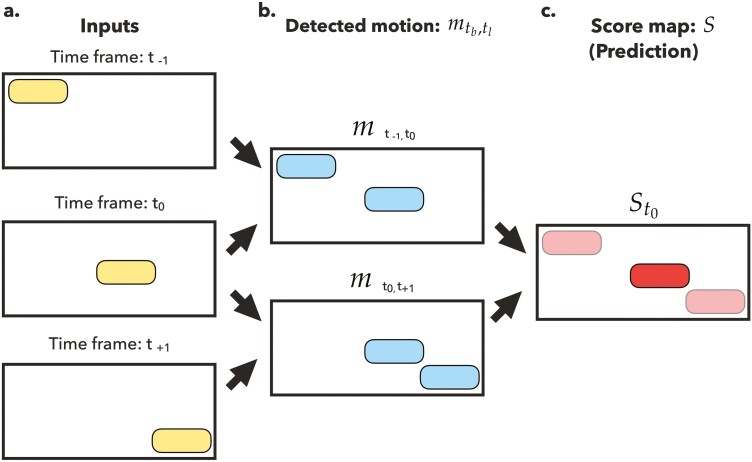
The computational approach to detect motions. (a) In this example, the input video containing three frames from t−1 to t+1. Yellow blocks represent the ground truths of animal positions. (b) Motions detected in the time range of ($t_{b}$,$t_{l}$). Blue blocks show the area with high motions. (c) The pixels with larger gradient of red means higher chance the areas are occupied by the studied objects.


Motion detection is useful to automatically propose potential pig positions for trackers. VTag will detect the pixels of interest (POI) over time frames, which are expected to cover entire animal bodies and help locate positions of pigs. The motion is quantified by the variation of pixel values in a time range of multiple neighboring time frames (Figure 1a). Assuming $p_{xyt}$ is a pixel value at coordinate x,y of the time framet, and $p_{xy\cdot }$ is an averaged value in the range ($t_{b}$,$t_{l}$) at coordinate x,y. The detected motion $m_{t_{b},t_{l}}\left(x,y\right)$ during the time range ($t_{b}$,$t_{l}$) is:

$$\begin{array}{l} \begin{array}{l} m_{t_{b},t_{l}}\left(x,y\right)=\frac{\underset{t\in \left(t_{b},t_{l}\right)}{\sum }\left(p_{xyt}-p_{xy\cdot }\right)}{t_{l}-t_{b}} \end{array} \end{array}$$

where $t_{b}$, $t_{l}$ are the beginning and the last time frames in the scanned time range, respectively. High pixel variation represents high motion values m for a pixel at coordinate x,y. If the studied pigs show up or leave out of the scope (pixel at (x,y)) in the middle of the time range ($t_{b}$,$t_{l}$), the pixel values therefore change from background colors to animal skin textures or from animal skin textures to background colors, resulting in high pixel variation (Figure 1b).

### Pig positions

By computing pixel motion for 2 flanking, equal-length time ranges, the positions of all monitored pigs can be located. For example, to infer the livestock positions at time frame $t_{0}$, pixel motion $m_{t_{-k},t_{0}}$ and $m_{t_{0},t_{k}}$ are calculated, where $t_{-k}$ and $t_{k}$ are k frames before and after the observed frame $t_{0}$, respectively. Then, summing 2 motion values can obtain a score map $S_{t_{0}}$ where high-value pixels are more likely to be occupied by the studied objects at the moment (Figure 1c). In this study, pixel scores $s_{t_{0}}\left(x,y\right)$ in the score map $S_{t_{0}}$ are defined with 2 frames before and after the observed frame $t_{0}$ as


$$\begin{array}{l} \begin{array}{l} s_{t_{0}}\left(x,y\right)=\underset{k=1}{\overset{2}{\sum }}\;\left(m_{t_{-k},t_{0}}\left(x,y\right)+m_{t_{0},t_{k}}\left(x,y\right)\right) \end{array} \end{array}$$

The scores s were hypothesized to follow a skewed distribution in which most pixels are observed with low scores. Hence, the 99th quantile of scores was set as the threshold to binarize pixels into 2 categories: POI is assigned a value of 1 if its score is greater than the threshold, otherwise value 0 is assigned to represent a background pixel. Finally, for each time framet, we have a binary map $B_{t}$ indicating the positions of the studied objects (i.e., pixel areas with non-zero values).

### Refining motion detection and proposals of tracking points

As the pig positions were inferred from observed motion, motion caused by irrelevant sources, such as human activities or pigs from other pens, should be avoided. Although the camera scope is limited to the studied pigs, some unrelated movements will be detected by our score functions and become noisy signals. For example, the noisy movements can be vibrations caused by the occasional collision between the pigs and pens or human activities. Such noisy signals are usually small in pixel areas and can be removed by “blurring” the binary map $B_{t}$ with a Gaussian kernel ω(Appendix) in convolutional operations. Each refined pixel $B{{\;}^{\prime }\;}_{t}\left(x,y\right)$ is:


$$\begin{array}{l} \begin{array}{ll} & B{{\;}^{\prime }\;}_{t}\left(x,y\right)=f\left(\omega *B_{t}\left(x,y\right)\right)\\ & =f\left(\underset{dx=-m}{\overset{m}{\sum }}\;\underset{dy=-n}{\overset{n}{\sum }}\;\omega \left(dx,dy\right)B_{t}\left(x+dx,y+dy\right)\right) \end{array} \end{array}$$

$$\begin{array}{l} f\left(x\right)=\left\{ \begin{array}{ll} 1, & if\;x\geq 0.5\\ 0, & otherwise \end{array}\right. \end{array}$$

where $B{{\;}^{\prime }\;}_{t}$ is the refined binary map in which POIs are represented by values of ones, and background colors are noted as zeros at the time framet. The lower and upper boundaries of the kernel ω are denoted by −m,m of *x*-dimension and −n,n of *y*-dimension, respectively. To save memory usage and avoid including noisy signals, we do not keep the positions of every POI but track their contours as representatives. We can obtain the finalized map $B^{\prime }{{\;}^{\prime }\;}_{t}$ containing the contours of objects with edge detection kernel γ(Appendix a) in the convolutional operation:


$$\begin{array}{l} B^{\prime }{{\;}^{\prime }\;}_{t}=\;\;\gamma \;\;*B_{t} \end{array}$$

Instead of keeping the entire area of POIs with high motion, the derived map $B^{\prime }{{\;}^{\prime }\;}_{t}$ only records the contours of POIs. The contours of POIs in $B^{\prime }{{\;}^{\prime }\;}_{t}$ are further clustered into object identities. A similarity matrix of each POI coordinate was calculated as a clustering constraint. With the defined constraint, agglomerative hierarchical clustering from the Python library, scikit-learn (Pedregosa et al., 2018), was performed to cluster POIs into each object identity. The centroids of each cluster are proposed as initial tracking points for the implemented trackers.

### Benchmark test

The implemented trackers were evaluated for their precision and computing time. To evaluate the precision, we manually labeled the central positions of each pig body as the ground truths. The precision error was determined by the Euclidean distance between the ground truth and the centroid of the predicted bounding box. To make the results comparable with other studies, the error was standardized by being divided by the diagonal distance of the video frame ranging from 0 to 1. In addition, as the tracking process may be unsuccessful when the similarity of two consecutive frames is low, human supervision is needed to provide new tracking positions to resume tracking. Hence, we also evaluated the number of supervision is needed to complete tracking the 300 frames in each dataset. To evaluate the computing time, the elapsed time to track one single frame is measured for 100 iterations. The time is presented in FPS by inverting the observed elapsed time. In addition to the implemented trackers, the object detection models, YOLOv5 and Mask R-CNN, pre-trained by the COCO dataset (Lin et al., 2014) are also included in the evaluation of computation time. It can help explore the possibility of adapting these pre-trained deep learning models in the pig tracking tasks. The evaluation was run on a personal laptop, MacBook Pro (14-in., 2021) with Apple M1 Max chip, 10 CPU cores, and 32 GB RAM. The GPU resources were not utilized during the evaluation.

## Results

### Software interface

**Figure 2.**
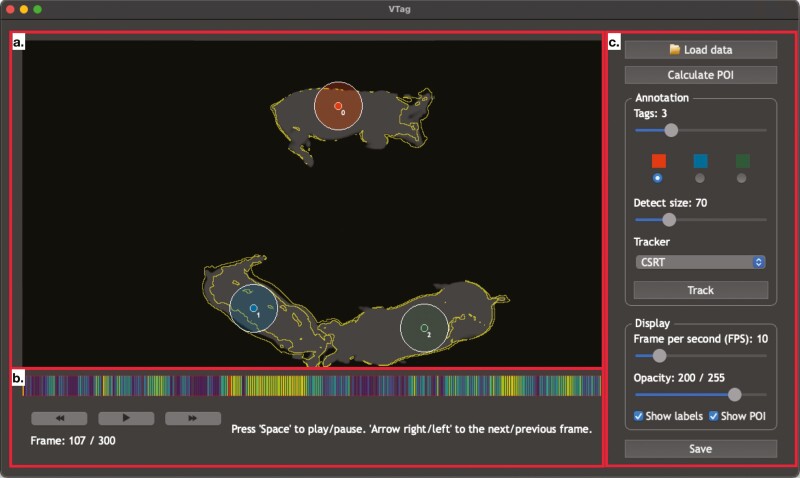
VTag graphical user interface. (a) Preview panel to display the tracking results. (b) Playback controller to traverse video frames and inspect tracking errors. (c) Configuration panel to fine-tune the tracking algorithm and import/export video data.

The VTag pipeline is released as a Python software and can be accessed by a GUI or an interactive Python session. There are 3 components that users can interact with in the GUI: the video previewer, the playback controller, and the configuration. The preview (Figure 2a) shows the video overlayed by the tracking results, which are presented by a centroid and its tracking window area. Different tracking points are colored differently to show pig entities. The video can be played, paused, and traversed to any video frame by interacting with the playback controller (Figure 2b). Each frame in the progress bar is colored in a gradient scale from yellow to blue, showing the tracking errors estimated from the implemented tracker. In the configuration panel (Figure 2c), parameters needed for the tracking task are tunable. In the panel, users can load a directory containing the video to start the tracking tasks, adjust the number of tracked objects and tracking size, and optimize the quality for displaying the tracking results. If users need to work with their own analysis in an interactive programming session, users can load VTag in Python as a library. The library has commands available that correspond to all the actions in the VTag GUI. In sum, VTag provides a friendly platform to annotate video data and generate informative farming guidance for pig activity.

### Benchmark testing

**Figure 3.**
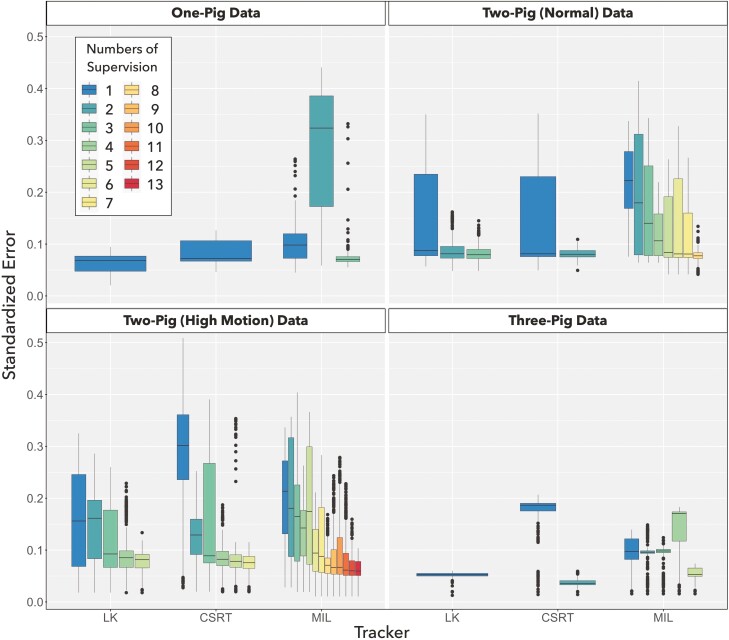
Evaluation of tracking precision. The standardized errors are plotted in box plots, which are colored in corresponding to the number of supervisions. Trackers, LK (sparse optical flow by Lucas and Kanade), CSRT (channel and spatial reliability), and MIL (multi-instance learning) are listed on the *x*-axis.

The precision evaluation is presented in Figure 3, the standardized errors over frames were plotted in boxplots. Every 0.1 of the standardized error is 26.29 cm in the presented datasets. The colors represent different supervisions. For example, the results shown in red are evaluated after 8 times of human supervision. With adequate human supervision, all trackers can precisely track pig activity with errors less than 22.82 cm in all the 4 datasets. In particular, the tracker LK can complete the tasks without any resuming supervision with the median errors of 18.03 and 13.81 cm for the datasets of one-pig and three-pig, respectively. The tracker CSRT performed similarly well with only one additional supervision with the median error of 16.3 cm in the studied datasets except the dataset of two-pig (high motion). Among the studied trackers, MIL has similar precision but requires more human supervision than others in all the dataset. It is noted that the number of tracked objects is not a major limiting factor when it comes to tracking precision. In this study, more supervision is needed when the objects are found to move rapidly and create blurry image features. When the pigs move rapidly, the input video with low FPS had latencies to display object positions timely. In the 2-pig dataset (high motion) although with similar precision, 7, 5, and 13 supervisions were needed to complete tracking the 300 frames for the 3 trackers, respectively.

**Figure 4.**
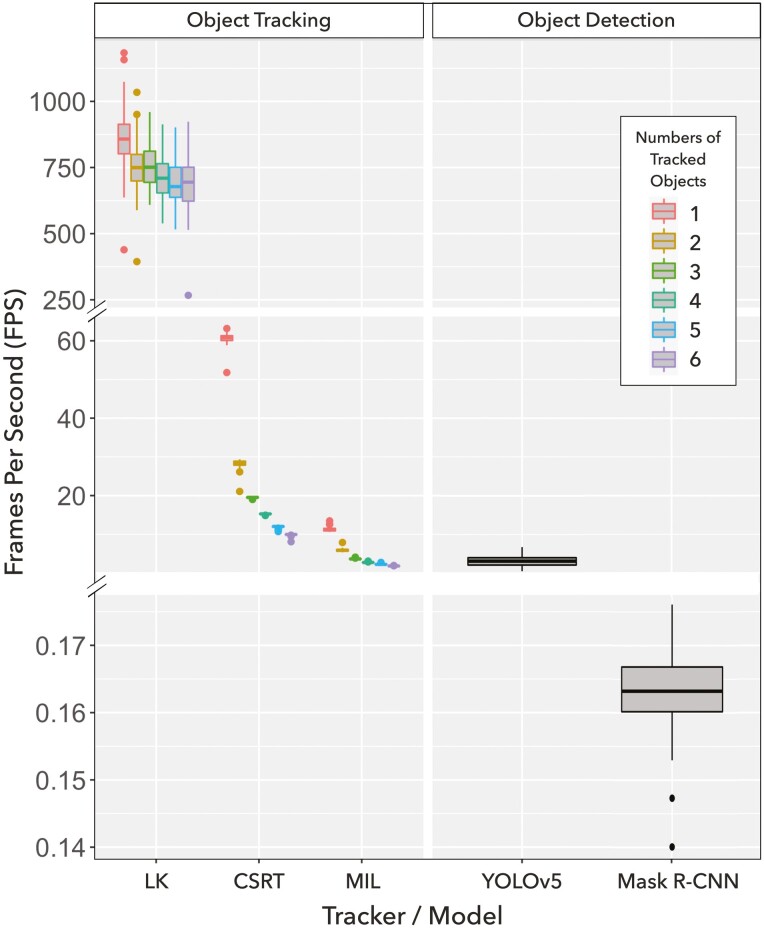
Evaluation of computing time by frame per second (FPS). FPS of each tracking task is plotted in box plots, which are colored corresponding to the number of tracked objects. Trackers, LK (sparse optical flow by Lucas and Kanade), CSRT (channel and spatial reliability), and MIL (multi-instance learning) are listed on the left column. The object detection models, YOLOv5 and Mask R-CNN, are plotted on the right column.

The computing time is presented by FPS, which indicates how many frames the tracker can process per second (Figure 4). As results, LK tracked averaged FPS of 900 and showed outperformance in computing speed to other trackers by more than 100 folds. CSRT is the second fast tracker with a performance ranging from 9.9 FPS to 60.81 FPS in the tasks of tracking different number of pigs. MIL is found to be the slowest tracker, with as low FPS as 1.8 FPS when it tracked six pigs. It is also found that for the trackers CSRT and MIL, the numbers of tracked objects affect the tracking speed nonlinearly. Additionally, the pre-trained object detection models are evaluated in this study as well. Without enabling GPU resources, both models predict the studied videos slower than the presented trackers. Only 4 FPS and 0.17 FPS are processed by YOLOv5 and Mask RCNN, respectively.

### Social interaction

**Figure 5.**
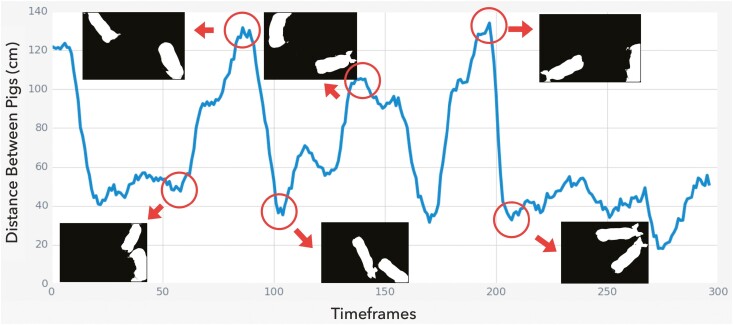
The predicted distances between pigs over all time frames in the two-pig data. The distance in centimeter is shown on the *y*-axis, and the *x*-axis represents the 300 timeframes. Six snapshots of the selected timeframes show the 2 extreme scenarios when pigs are closely engaged or separated apart.

The distance between studied subjects implies 2 types of general social interactions: separated or engaged. When the subjects engage closely, the distance values are low during the period of time frames. Otherwise, subjects are separated apart without much interaction. A line chart of the distance against the 300 timeframes was visualized to monitor such patterns, showing 4 peaks and 4 valley values from the 2-pig data (Figure 5). To examine whether the distance is an effective indicator for the interactions, video frames with peak and valley values were displayed. Consequently, in the frames with peak values, interaction was observed among pigs, and they were observed staying in 2 different corners of the pen at the examined time frame. On the other hand, in the frame with valley values, social interactions were observed for all inspected frames. Pigs were in the status of in-taking feeds alongside or chasing each other. From the examined 300 frames, the estimated distance between pigs is an accurate indicator to filter time frames where social interactions may occur.

**Figure 6.**
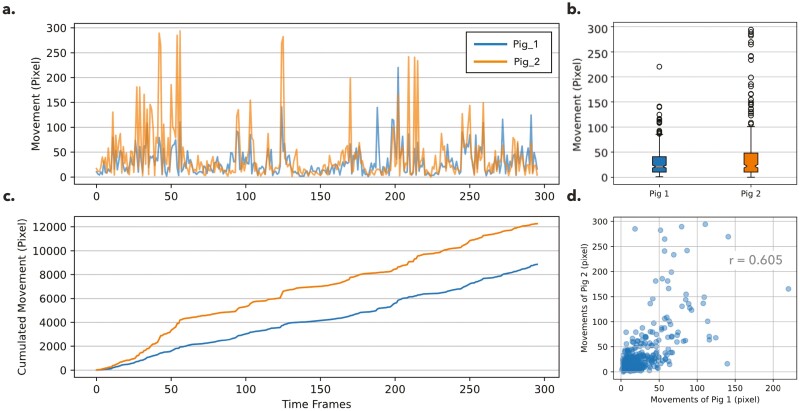
The movement of pigs in pixels in the two-pig dataset. Two pigs are colored by blue and orange. (a) A line chart showing the pixel movement (*y*-axis) per time frame (*x*-axis). (b) A boxplot comparing the movement between two pigs. (c) A line chart showing the accumulated pixel movement (*y*-axis) per time frame (*x*-axis). (d) A correlation plot between the movement of Pig 1 (*x*-axis) against Pig 2 (*y*-axis), and each dot represents the movements of the observed time frame.

By knowing the tracks of each pig, pixel movements per time frame were studied to monitor the activities individually. In the presented data, 2 studied pigs were denoted as “Pig_1” and “Pig_2”. The median movement of Pig_1 and Pig_2 is 21.1 and 21.98 pixels per frame, which show no significant difference (*P*-value = 0.953) in overall activity (Figure 6a and b). However, individual-specific temporal pattern can be discovered by dissecting the activity at certain time frames. For example, during the first 50 frames, Pig_2 was much more active, the difference between Pig_1 and Pig_2 was especially revealed in those peak movements. Moreover, after the 50th frame, Pig_2 continuously had greater changes of accumulated movements over Pig_1. The superiority was 1739.7 pixels at the 50th frame, and it was later expanded to 3612.9 pixels at the 250th frame (Figure 6c). Finally, we inspected the synchronicity between pigs by comparing their movements per frame (Figure 6d). A moderate correlation (*r* = 0.605, *P*-value < 0.01) was observed in the studied data, which implied that the activity of each individual was not independent and were partially determined by its neighboring pig.

**Figure 7.**
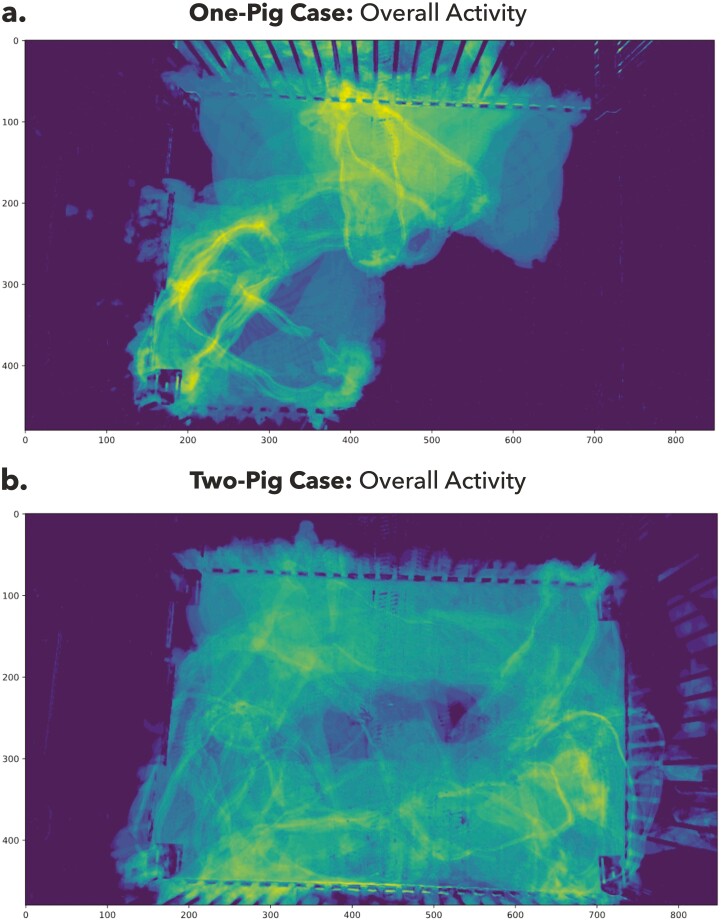
Heatmaps showing the spatial activity of pigs. The yellow areas are visited the most by the pigs, and the purple area indicates the least visited region in the view scope. (a) One-pig dataset; (b) two-pig dataset.

In addition to monitoring the temporal activity, spatial patterns of pig movement can be informative for herd management. Heat maps (Figure 7) generated from pixel-wise variation across all time frames provided insightful guide on what areas were visited most (coded in yellow). In the one-pig data (Figure 7a), middle-top and bottom-left regions have found to be the hot spots, which were the places to engage with neighboring pigs and the feeding area, respectively. Whereas in the 2-pig data (Figure 7b), there was no clear spatial trend of the subject activity. Most corners of the pen were visited by both pigs except the central area.

## Discussion

Continuously tracking pig activity from videos is an important initial step to monitor farming conditions in swine industry. Including animal diseases, welfare, and pen-scale social interactions, such complex monitoring tasks require detailed observation of pig activity. Many existing works have automated the tasks through the aid of CV technology but required massive human effort in preparing data sets to build an effective system. In contrast, this paper presented a semi-supervised pipeline, VTag, which does not require laborious work in setting up the training system. Solely relying on a top-view and grayscale video, VTag provides an efficient approach to continuously track the positions of group-housed pigs with an average error of 17.99 cm in the presented datasets. The results can serve as preliminary farming guidance to infer complex traits that used to require intensive labor resources.

For example, by continuously tracking pig positions with VTag, individual-level activity per unit time and walking speed can be estimated. This is important information for the trait assessment of pig lameness, which can be potential indicators of fractures, lesions, and development disease (Heinonen et al., 2013; Benjamin and Yik, 2019), and diminishes welfare in pigs. Hence, effectively evaluating lameness allows farmers to control economic losses from losing pigs with poor body conditions (Anil et al., 2009). Another important monitoring task that can be improved with VTag is tail biting in pigs. Because tail biting is linked to stressful farming conditions (D’Eath et al., 2014) and lower body weights (Marques et al., 2012), detecting the negative events at an early growing stage can be beneficial to both animal welfare and production. As the real-time pig positions are obtained automatically, the relative distance between individuals in the pen can be estimated. Behavioral researchers can use this information to filter a specific time range from an hour-long video: When the relative distance is low, it is more likely to observe tail-biting events.

In addition to evaluating pig behaviors through RGB videos, automation can be further facilitated by other sources. For example, videos containing depth information are useful to estimate pig body weights. Body weight is a critical trait associated with growing rate, feed efficiency, and meat biomass. Conventionally, pigs are weighed on the electronic scale in the pen, but it can be either inaccurate when more than one pig is standing on the scale or expensive if the scale is integrated into the feeding system. A past study has presented a video-based pipeline that can successfully estimate pig body weight with an RGB-depth camera by segmenting pig contours (Yu et al., 2021). By combining the existing work and VTag, which can continuously track pig positions, the fully automatic system of pig weighing is feasible for farms with limited resources.

The implemented trackers have successfully shown their great performance of tracking objects in their published papers (Babenko et al., 2009; Lukežič et al., 2018). However, the trackers failed to track pigs without any human supervision in our presented results. The potential reasons may be explained by the difference in the monitoring context. In our study, the tracked objects share similar morphological features. Even for the feature-rich areas, such as pig heads and tails where unique spatial patterns are observed, they were hard for trackers to distinguish the difference between different pig individuals. The trackers easily lost tracking the correct individual when two pigs frequently interact with each other in a short period. Another reason is the video quality. In the papers where the trackers were published, the demonstrated videos recorded at least 20 FPS. Whereas the studied datasets only have 6 FPS, which is a common setting in practical farming to reduce power dissipation and save data storage. Such low FPS videos reduce the similarity between consecutive frames and increase the chance of mismatching tracked features over time (Porikli and Tuzel, 2005; Li et al., 2007). Additionally, when the tracked object moves rapidly, the track features are more likely to become blurry in a low-FPS video. These limitations make the tracking task in livestock farming more difficult than the regular tracking task, where videos have 30 FPS, and the video frames are assumed to be similar in adjacent frames and the tracked features are unique compared to other objects.

We also included pre-trained models, YOLOv5 and Mask R-CNN, in our benchmark study. The low-FPS results indicate that it is difficult to fulfill real-time long-term monitoring in livestock farming without accessing GPU resources. Although we did not show their precision in the current work, the detection results are not comparable with the presented trackers. It is because the models were trained by COCO datasets, in which top-view pig images are not included. In some video frames, pigs are either not detected, or 2 adjacent pigs are identified as the same object. Besides, without further modification of the models, it cannot force tracking the certain number of objects. These limitations make the evaluation difficult when we want to compare the precision of tracking the same number of pigs. In conclusion, the results suggest that the object detection models are not as suitable as object tracking algorithms in the pig monitoring tasks.

Further improvement can be made in the current version of VTag. For example, VTag is found to mis-identified pig identities when individuals frequently contact each other in a short time as described earlier. Although the wrong labels can be corrected manually, it still requires time and effort from humans’ supervision. One way to reduce such error is to utilize a strategy called template learning, which was discussed in the literature (Lan et al., 2017; Wang et al., 2019). The general idea of this strategy is to first select the video frames in which pigs are not in close range of their neighbors. Then, the pig morphology observed in the selected frames is learned as “templates”. Finally, the model can use the templates to update the predictions in the frames where pig positions are mis-identified due to the closed distance between pigs.

In addition to improving the algorithm, adding information by wearable devices is also helpful to increase the monitoring precision. The devices, including motion sensors, magnetometers, gyroscopes, and GPS receivers, have been widely used to monitor behavioral patterns in large farming environments (i.e., pastures and barns) (Chapa et al., 2021; Li et al., 2021; Nikodem, 2021; Perisho and Hajnal, 2021; dos Reis et al., 2021). Specifically, with the use of tracking collar wore by pastured livestock, grazing behaviors were successfully identified for cattle (Brennan et al., 2021) and sheep (dos Reis et al., 2021). The spatial resolution for outdoor studies was further improved to centimeter-level by coupling sensor collars with signal receivers deployed around the farm (Li et al., 2021). In group-housed scenarios, Smartbow (Weibern, Austria), a commercialized ear-tag sensor system, also demonstrated promising results in monitoring complex interactions on reproduction traits in swine cohorts (Perisho and Hajnal, 2021) and feeding behaviors of cows (Chapa et al., 2021). In conclusion, by coupling the VTag algorithm and the described improvement, the automation of the assessment system is expected to monitor more complex farm settings.

## Conclusion

The presented semi-supervised pipeline, VTag, can track pigs in the video clips with a minimal human supervision to achieve decent precision. Among the tested trackers, a simple algorithm as spare optical flow can achieve ideal balance between precision and computing speed for the pig tracking tasks. The observed median error in the studied videos is no larger than 17.99 cm in predicting positions of each individual. Such performance provides individual-level guidance for farming management in knowing animal activity, visiting hot spots, and social interaction. In conclusion, this study reports a rapid, precise deployment of high-throughput assessment for continuous pig tracking. With the efficient monitoring system, animal health and derived products can be greatly improved.

## Abbreviations

CV: computer vision
GPU: graphics processing unit
POI: pixels of interest
RGB: red, green, and blue
VTag: virtual tag

## Appendix

$$\begin{array}{l} Gaussian\;kernel\;\omega \;=\;\left[\begin{array}{ccc} 1 & 4 & 1\\ 4 & 9 & 4\\ 1 & 4 & 1 \end{array}\right] \end{array}$$

$$\begin{array}{l} Edge\;detection\;kernel\;\gamma \;=\;\left[\begin{array}{ccc} -1 & -1 & -1\\ -1 & 8 & -1\\ -1 & -1 & -1 \end{array}\right] \end{array}$$
